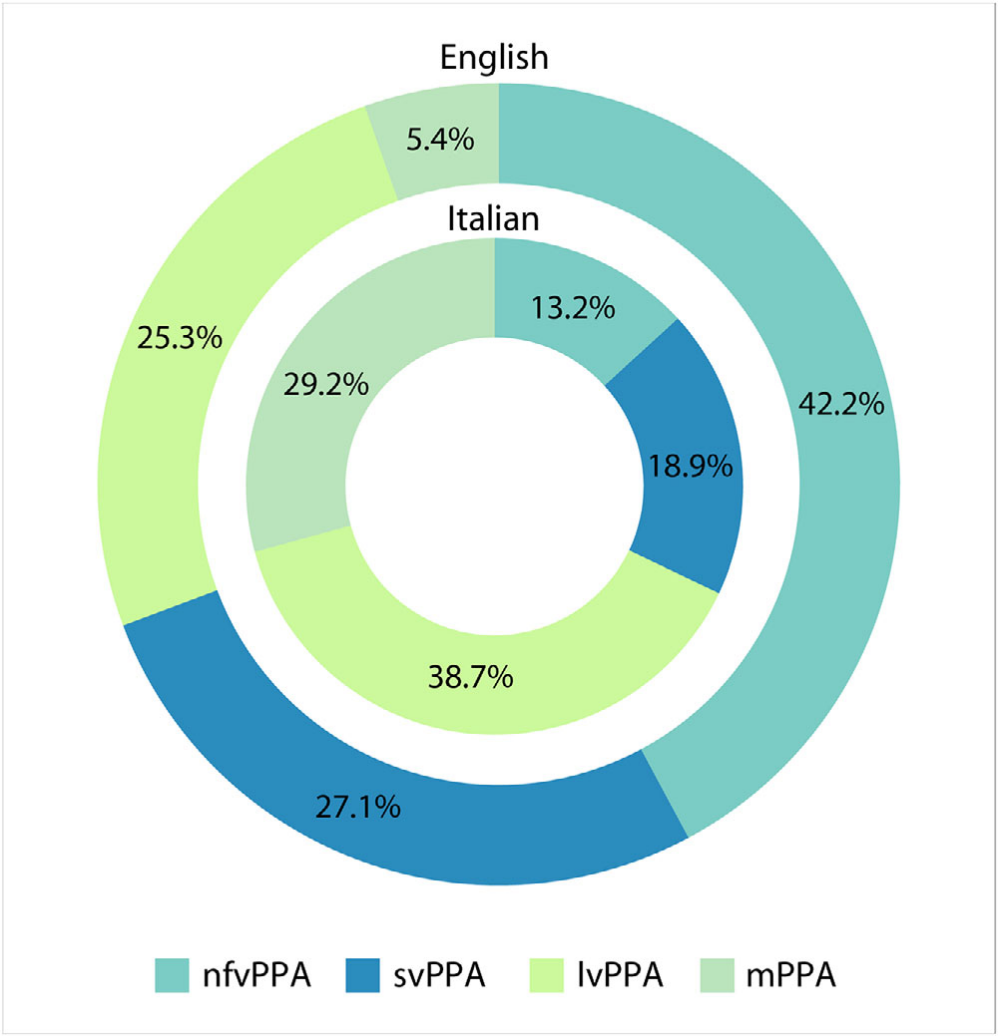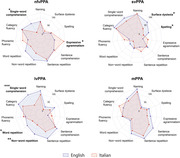# Primary progressive aphasia in Italian and English: a cross‐linguistic cohort study

**DOI:** 10.1002/alz.085933

**Published:** 2025-01-03

**Authors:** Salvatore Mazzeo, Chris JD Hardy, Jessica Jiang, Carmen Morinelli, Valentina Moschini, Jeremy Johnson, Anthipa Chokesuwattanaskul, Anna Volkmer, Jonathan D. Rohrer, Assunta Ingannato, Silvia Bagnoli, Sonia Padiglioni, Benedetta Nacmias, Sandro Sorbi, Valentina Bessi, Jason D Warren

**Affiliations:** ^1^ Vita‐Salute San Raffele University, Milan Italy; ^2^ Dementia Research Centre, UCL Queen Square Institute of Neurology, University College London, London United Kingdom; ^3^ Department of Neuroscience, Psychology, Drug Research and Child Health, University of Florence, Florence Italy; ^4^ Dementia Research Centre, Department of Neurodegenerative Disease, UCL Queen Square Institute of Neurology, University College London, London United Kingdom; ^5^ Research and Innovation Centre for Dementia‐CRIDEM, AOU Careggi, Florence, Florence Italy; ^6^ Dementia Research Centre, Queen Square Institute of Neurology, University College London, London United Kingdom; ^7^ King Chulalongkorn Memorial Hospital, Thai Red Cross Society, Bangkok Thailand; ^8^ Cognitive Clinical and Computational Neuroscience Research Unit, Faculty of Medicine, Chulalongkorn University, Bangkok Thailand; ^9^ Department of Psychology & Language Sciences, University College London, London United Kingdom; ^10^ Department of Neuroscience, Psychology, Drug Research and Child Health, University of Florence, Florence, Florence Italy; ^11^ IRCCS Fondazione Don Carlo Gnocchi, Florence, Florence Italy

## Abstract

**Background:**

Primary Progressive Aphasia (PPA) is a neurodegenerative disorder primarily affecting language abilities, with clinical variants (nonfluent/agrammatic variant [nfvPPA], semantic variant [svPPA], logopenic variant [lvPPA], and mixed‐PPA [mPPA]) categorized based on linguistic features. This study aims to compare PPA cohorts of native speakers of two different languages: English (an analytic language with deep orthography) and Italian (a synthetic language with shallow orthography).

**Methods:**

We considered 166 English participants (70 nfvPPA, 45 svPPA, 42 lvPPA, 9 mPPA) and 106 Italian participants (14 nfvPPA, 20 svPPA, 42 lvPPA, 31 mPPA). Starting from the neuropsychological battery used to assess patients, we extracted one test for each cognitive and linguistic function that can be compared between cohorts. Comparisons were adjusted for symptom duration and Mini‐mental State Examination scores.

**Results:**

The English cohort included a higher proportion of nfvPPA patients (42% vs. 13%, p<0.001), while the Italian cohort showed higher proportions of lvPPA (25% vs. 38%, p = 0.019) and mPPA (5% vs. 29%, p<0.001). English nfvPPA patients were more frequently impaired in single‐word comprehension (60% vs. 8%, p = 0.013), while Italian nfvPPA patients exhibited more agrammatism (46% vs. 93%, p = 0.015). English svPPA had a higher proportion of surface dyslexia (68% vs. 30%, p = 0.046) and spelling impairment (38% vs. 10%, p = 0.021). English lvPPA had broader impairments, including single‐word comprehension (89% vs. 29%, p<0.001), repetition of words (61% vs. 26%, p = 0.03), nonverbal working memory (69% vs. 36%, p = 0.005), and visuospatial perception (89% vs. 25%, p<0.001).

**Conclusions:**

This study reveals linguistic and cognitive distinctions between English and Italian PPA cohorts, emphasizing the impact of language‐specific characteristics on symptomatology. Cultural and linguistic nuances should be considered in PPA diagnosis and management, calling for more tailored assessments and criteria.